# Effects of Gestational Age at Birth on Cognitive Performance: A Function of Cognitive Workload Demands

**DOI:** 10.1371/journal.pone.0065219

**Published:** 2013-05-24

**Authors:** Julia Jaekel, Nicole Baumann, Dieter Wolke

**Affiliations:** 1 Department of Developmental Psychology, Ruhr-University Bochum, Bochum, Germany; 2 Department of Psychology, University of Warwick, Coventry, United Kingdom; 3 Warwick Medical School, University of Warwick, Coventry, United Kingdom; The Ohio State University, United States of America

## Abstract

**Objective:**

Cognitive deficits have been inconsistently described for late or moderately preterm children but are consistently found in very preterm children. This study investigates the association between cognitive workload demands of tasks and cognitive performance in relation to gestational age at birth.

**Methods:**

Data were collected as part of a prospective geographically defined whole-population study of neonatal at-risk children in Southern Bavaria. At 8;5 years, *n* = 1326 children (gestation range: 23–41 weeks) were assessed with the K-ABC and a Mathematics Test.

**Results:**

Cognitive scores of preterm children decreased as cognitive workload demands of tasks increased. The relationship between gestation and task workload was curvilinear and more pronounced the higher the cognitive workload: GA^2^ (quadratic term) on low cognitive workload: *R* ^2^ = .02, *p*<0.001; moderate cognitive workload: *R* ^2^ = .09, *p*<0.001; and high cognitive workload tasks: *R* ^2^ = .14, *p*<0.001. Specifically, disproportionally lower scores were found for very (<32 weeks gestation) and moderately (32–33 weeks gestation) preterm children the higher the cognitive workload of the tasks. Early biological factors such as gestation and neonatal complications explained more of the variance in high (12.5%) compared with moderate (8.1%) and low cognitive workload tasks (1.7%).

**Conclusions:**

The cognitive workload model may help to explain variations of findings on the relationship of gestational age with cognitive performance in the literature. The findings have implications for routine cognitive follow-up, educational intervention, and basic research into neuro-plasticity and brain reorganization after preterm birth.

## Introduction

The human brain is highly susceptible to the consequences of preterm birth [1], [2]. Depending on the timing and severity of gestational insults, the functional architecture may be substantially altered to affect overall cognitive development [3]–[5]. Prematurity is associated with alterations in brain development (i.e. brain insult [5] and reduced brain volume [6], [7]), white matter microstructure [8], cortical folding [9], and the thalamic system [10]. These reorganizations of cortical and neurological structures after preterm birth are still detectable in childhood and adolescence in multiple regions [11], [12].

Very preterm children score lower on overall cognitive performance, have more often multiple cognitive problems and more often specific deficits in mathematic tasks than full term children [13], [14]. Some have reported that these deficits are already detectable in late or moderately preterm children [15], [16]. Although children born between 32 and 36 weeks gestational age account for 5–10% of all births their long-term sequelae have only recently started to attract attention [17]. In particular, cognitive problems of preterm children seem to affect their ability to perceive, integrate, and process stimuli simultaneously [3], i.e. solve more complex tasks [18]. Accordingly, it has been suggested that cognitive tasks involving simultaneous processing of information (e.g. visuospatial pattern recognition) may be more affected by preterm delivery than tasks involving sequential processing (e.g. digit recall one after the other) [19]–[21]. The aim of this study was to investigate the association of task complexity and thus cognitive workload requirements in relation to cognitive performance by gestational age.

Working memory models suggest that cognitive resource utilization increases with task complexity to allow for adequate behavioral performance even in the most demanding situations [22], [23]. Specifically, it has been proposed that cortical areas are specialized for certain tasks but each cortical area has limited computational capacities restraining its activity [24]. With increasing cognitive workload of a task more resources are needed and thus more cortical areas (i.e. large scale cortical networks) are recruited [24]. Accordingly, a “cognitive control network” may be involved that coordinates allocation of brain resources [25].

Considering the findings of altered brain development and superimposed injury to the brain according to degree of prematurity [26] we speculate that computational capacities of individual brain areas may be more limited with decreasing gestational age at birth. This effect of gestation may become more apparent the higher the cognitive workload of a task. Considered within the cognitive workload model described above, we successfully tested the following hypotheses:

1. The higher the cognitive workload of tasks, the larger the performance deficits with decreasing gestation.

2. This relationship between gestation and cognitive task workload is curvilinear with disproportionally higher deficits in performance for very preterm children the higher the cognitive workload of the task [15], [27].

3. Biological factors that are related to brain development such as gestation, birth weight, and neonatal complications explain more of the variance in performance in high compared with low or moderate cognitive workload tasks within different gestation groups.

## Methods

### Ethics statement

Ethical permission for the study was granted by the Ethics committee of the University of Munich Children's Hospital and the Bavarian Health Council (Landesärztekammer). Participating parents were approached within 48 hours of the infant's hospital admission and were included in the study once they had given written consent for their child to participate.

### Participants

Data were collected as part of the prospective Bavarian Longitudinal Study (BLS) [20], [28]. The BLS is a geographically defined whole-population sample of children born between January 1985 and March 1986 within a geographically defined area of Southern Bavaria (Germany) who required admission to a children's hospital within the first 10 days of life (*n* = 7505; 10.6% of all live births). Additionally, 916 healthy control infants (normal postnatal care) were identified at birth from the same hospitals in Bavaria during the same period (Phase I).

Of the initial sample, *n* = 255 survivors born <32 weeks of gestation and a subsample of the children born >31 weeks of gestation (*n* = 1071, randomly drawn within the stratification factors gender, socio-economic status (low, moderate, and high SES), and degree of neonatal risk (none, low, moderate, high, very high)) were assessed with cognitive tests at 8;5 years of age. Full details of the sampling criteria and dropout rates are provided elsewhere [20], [29]. For this study, the characteristics of the initial total population sample were compared with the sample of participants assessed at 8;5 years (*n* = 1326) according to gestational age groups. In order to achieve representativeness of the final study sample, the cases in the early term and full term gestation groups were weighted according to neonatal biological risk (please see Table S1). Table 1 shows the characteristics of the final weighted sample.

**Table 1:** Sample characteristics of the weighted BLS Phase II study participants according to gestational age groups (cases with severe neurological impairment excluded) at 8;5 years.

		<32 w GA	32–33 w GA	34–36 w GA	37–38 w GA	39–41 w GA
		*n* = 255	*n* = 90	*n* = 209	*n* = 186	*n* = 586
GA		29.55 (1.59)	32.52 (0.50)	35.10 (0.76)	37.52 (0.50)	39.92 (0.67)
Birth weight		1294 (348)	1656 (379)	2207 (558)	2809 (562)	3364 (524)
OPTI score^1^		9.55 (2.66)	7.92 (2.60)	5.48 (2.83)	3.61 (2.53)	2.63 (2.14)
Ventilation (duration/days)		19 (23)	5 (9)	1 (4)	0 (2)	0 (1)
Hospitalization (days)		83 (41)	53 (22)	29 (20)	16 (16)	12 (19)
Neonatal risk score						
none		0%	0%	5%	14%	17%
low		0%	2%	17%	27%	35%
moderate		0%	23%	31%	38%	34%
high		0%	74%	46%	21%	14%
very high		100%	0%	0%	0%	0%
Child sex (male)		57%	48%	51%	48%	50%
Family SES	low	36%	35%	35%	34%	35%
medium		43%	37%	29%	31%	25%
high		21%	28%	36%	35%	40%

1 Higher OPTI scores indicate less optimal neonatal course. Data is presented as mean (*SD*) for continuous variables and percentages (*%*) for categorical variables.

### Measures

#### Biological variables

Gestational age (GA) was determined from maternal reports of the last menstrual period and serial ultrasounds during pregnancy. When the estimates of these two differed by more than two weeks, postnatal Dubowitz scores were used [30]. Birth weight was documented in the birth records. Infant postnatal complications were assessed with a comprehensive optimality index (OPTI) including 21 items (e.g. ventilation or intubation, severe anaemia, cerebral haemorrhage) [31]. Infant neonatal risk (Intensity of Neonatal Treatment Index, INTI) [29] was computed from daily ratings of care level, respiratory support, feeding dependency, and neurological status during the initial hospitalization. This INTI score was recoded into five risk categories (0 = none, 1 = low, 2 = moderate, 3 = high, 4 = very high risk) and used as stratification variable of the BLS Phase II sample of children (Table 1).

#### Family socio-economic background (SES)

Information was collected through structured parental interviews within 10 days of child birth. Family SES was computed as a weighted composite score derived from the occupation of the self-identified head of each family together with the highest educational qualification held by either parent [32].

#### Cognitive assessments

At 8;5 years of corrected age, children's cognitive abilities were assessed with the German version of the Kaufman Assessment Battery for Children, K-ABC [33], [34]. In addition, to assess numerical representations and reasoning, children were administered a mathematics test [28], [35]–[37]. Test tasks were presented to children in book form with 29 items assessing numerical estimations, reasoning, and mental rotation abilities. Item responses were scored for accuracy and subscale scores were summed into a total score. All cognitive assessments were carried out by trained assistant psychologists that were blind to children's background characteristics.

#### Cognitive workload of cognitive tests

Cognitive tasks were ordered theoretically according to their within working memory manipulation of integrating information (i.e. sequential vs. simultaneous [21], [33], [38]; low, intermediate, high [23]; variance shared with a *g* factor of intelligence [22], [23]). This order was confirmed statistically with a principal component analysis (PCA) on the test scores of the healthy full term control children within the sample to reveal each task's loading on *g* (high workload tasks require integration of various cognitive processes thus intercorrelations among these tasks should be higher than among low workload tasks [23], please see Table 2 for details).

**Table 2:** K-ABC subtests' and Mathematic Test's cognitive workloads according to different models.

Test	Task name	Task description	Kaufman model [33]	MDS spatial model [23]	Working memory as *g*-factor	
					Subtest variance shared with *g* [23]	PCA within healthy control sample (*n* = 312)
K-ABC	Number recall^a^	Repetition of a number of digits	sequential	low	.34	.48
K-ABC	Hand movements^a^	Performance of a series of hand movements	sequential	intermediate	.53	.50
K-ABC	Word order	The child is asked to touch silhouettes of common objects as named by the tester	sequential	intermediate	.59	.69
K-ABC	Gestalt closure^b^	Naming of an object pictured in a partially completed drawing	simultaneous	intermediate	.58	.41
K-ABC	Matrix analogies^b^	Selection of a picture that completes a visual analogy	simultaneous	intermediate	.63	.59
K-ABC	Triangles	Assembly of identical triangles into an abstract pattern that matches a model	simultaneous	intermediate	.60	.62
K-ABC	Spatial memory	Recall of the location of pictures on a page previously presented	simultaneous	low	.47	.48
K-ABC	Photo series	Chronological ordering of photographs of an event	simultaneous	intermediate	.67	.55
K-ABC	Riddles	The tester describes characteristics of a verbal concept and the child names it	achievement/reasoning	high	.84	.69
K-ABC	Arithmetic^c^	The child is asked to solve arithmetic problems	achievement/reasoning	high	.80	.71
Maths Test	Index score^c^	29 items assessing numerical estimations, reasoning, and mental rotation abilities	-	-	-	.70

a incorporated in the low cognitive workload scale;

b incorporated in the moderate cognitive workload scale;

c incorporated in the high cognitive workload scale.

Accordingly, we selected six prototypical test tasks that required low (K-ABC Number recall, K-ABC Hand movements), moderate (K-ABC Gestalt closure, K-ABC Matrix analogies), or high cognitive workload (K-ABC Arithmetic, Mathematics Test) for further analysis.

### Statistical analyses

**Figure 1:**
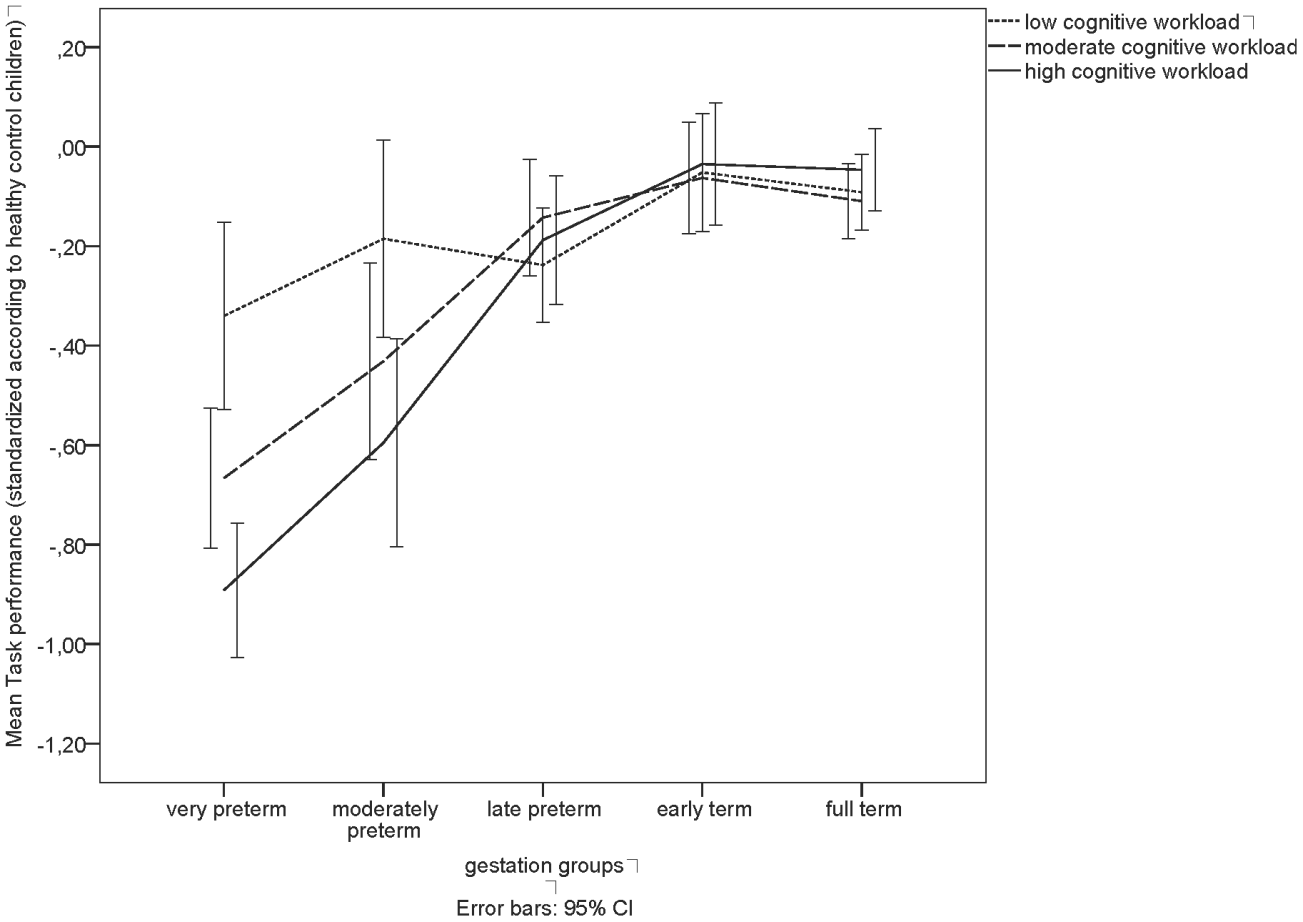
Task performance according to workload and gestational age. Bars represent children's mean task performance (*z*-scores +/−95% confidence intervals (*CI*)) at age 8;5 years on low, moderate, and high cognitive workload tasks according to different gestational age groups (*n* = 1326).

**Figure 2:**
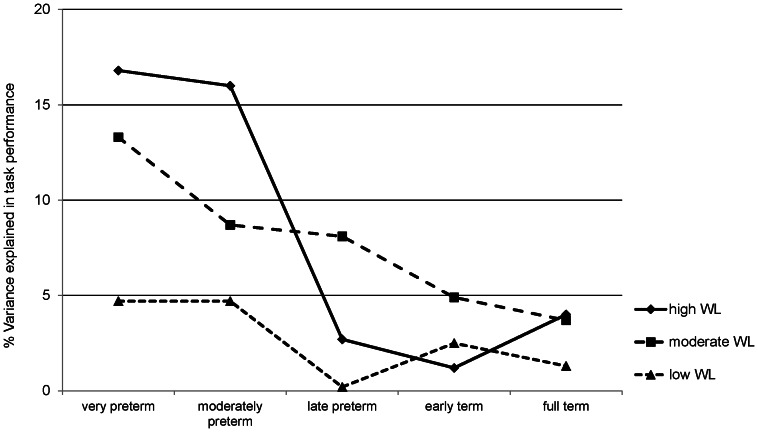
Variance explained by biological factors according to workload and gestational age. Symbols represent the unique percentage of variance explained (*R* ^2^) by biological factors (GA, birth weight, OPTI score) in children's performance on low, moderate, and high cognitive workload (WL) tasks at age 8;5 years according to gestational age groups (*n* = 1326).

Firstly, the scores of all participants were *z*-standardized according to the mean scores of the healthy control children within the sample (*n* = 312; mean GA = 39.43 (*SD* = 1.38), neonatal risk score  =  0). Analyses were performed on a weighted, population-representative sample (see Table S1) in order to insure that the degree of neonatal risk for each GA group was similar to that for all of the children in the respective groups recruited at birth. Secondly, we computed mean values of task performance (including 95% confidence intervals) by GA groups (very preterm: <32 weeks GA; moderately preterm: 32–33 weeks GA; late preterm: 34–36 weeks GA; early term: 37–38 weeks GA; full term 39–41 weeks GA) according to cognitive workload (low, moderate, and high, respectively) (Figure 1). We then used curve estimation analysis in order to identify the best fitting linear or curvilinear function for the effect of gestation on task workload. Thirdly, we conducted a multivariate analysis of variance (MANOVA) to scrutinize the effect of gestational age on children's performance in low, moderate, and high cognitive workload tasks (Table 3). Finally, we investigated how much variance was explained by early biological factors (GA, birth weight, OPTI score) in high, moderate and low workload tasks within the different gestation groups (Figure 2).

**Table 3:** Multivariate analysis of variance (*MANOVA*) testing the effects of gestational age on children's performance in low, moderate, and high cognitive workload tasks (*n* = 1326).

Predictor	Pillai's Trace	*F*	*df*	*p-Value*
Gestational age (GA)	.08	28.34	3, 934	<0.001
GA^2^	.09	29.24	3, 934	<0.001
Interaction of GA^2^ with low workload	.95	6011.89	3, 934	<0.001
Interaction of GA^2^ with moderate WL	.93	4027.56	3, 934	<0.001
Interaction of GA^2^ with high WL	.93	3932.09	3, 934	<0.001

## Results

### Cognitive performance as a function of gestation and cognitive workload

Figure 1 shows *z*-standardized mean values of children's task performance (with 95% confidence intervals) by gestational age groups according to cognitive workload. Disproportionally higher deficits in performance for very and moderately preterm children were found with increasing cognitive workload of the tasks.

Curve estimation analysis revealed that the best fitting functions for the effect of gestation on task performance were quadratic (GA^2^ (quadratic term) on low cognitive workload: *R* ^2^ = .02, *F* = 10.06, *p*<0.001; moderate cognitive workload: *R* ^2^ = .09, *F* = 46.06, *p*<0.001; and high cognitive workload tasks: *R* ^2^ = .14, *F* = 77.45, *p*<0.001, respectively). To further scrutinize this effect of gestational age on children's performance we conducted a multivariate analysis of variance (MANOVA) with the low, moderate, and high cognitive workload tasks as dependent variables. Table 3 shows that gestational age (GA) and GA^2^ (quadratic function) as well as the interactions of GA^2^ with low, moderate, and high cognitive workload significantly predicted performance.

We then tested how much of the variance in task performance was explained by early biological factors (GA, birth weight, OPTI score). Regression analyses revealed that the percentage of variance explained in performance by biological factors (*R* ^2^) increased from 1.7% (*F* = 7.55; *p*<0.001) in low cognitive workload tasks to 8.1% (*F* = 38.83; *p*<0.001) in moderate cognitive workload tasks, and 12.5% (*F* = 62.62; *p*<0.001) in high cognitive workload tasks.

Finally, we examined within each gestational age group how much of the variance in low, moderate, and high cognitive workload tasks was explained by biological factors. Figure 2 shows that the percentage of variance explained by biological factors was highest in high cognitive workload tasks among very and moderately preterm children (16.8% and 16.0%, respectively) whereas it was lowest in low cognitive workload tasks among children born at 34 weeks GA and older (0.2% to 2.5%, respectively).

## Discussion

The aim of this study was to investigate the association between cognitive workload and cognitive performance according to gestational age at birth. We tested an adapted cognitive workload model that may help to explain how prematurity affects brain reorganisation and thus functional cognitive performance. The results support our hypotheses regarding cognitive workload and cortical resources: Firstly, we found that performance deficits of preterm children increased as cognitive workload of tasks increased. Secondly, this relationship between gestation and task workload was curvilinear with disproportionally higher deficits in performance for very and moderately preterm children (<34 weeks GA) the higher the cognitive workload of the tasks.

Previous studies have shown that visuospatial working memory is particularly impaired in very preterm children [39]. Indeed, the two visuospatial tasks (K-ABC Gestalt closure and Matrix analogies) required moderate cognitive workload. However, the two mathematics tests assessing conceptual reasoning (not achievement) required the highest cognitive workload (K-ABC Arithmetic and Mathematics Test). Thus, consistent with recent findings of particular impairment in mathematical performance of very or extremely preterm children [14], [36], [40] our results suggest that the cognitive complexity of mathematical estimations and reasoning may pose the largest challenge to the preterm brain's computational capacities. Compared with other models of cognition or executive functions [41], [42], the cognitive workload model may provide a useful alternative to design or select tasks that measure specific effects of low GA as it integrates theoretical and practical approaches of multiple domains (i.e. neuroscience, cognitive modelling, paediatrics, and developmental psychology).

Alterations in the functional architecture of the brain aggravate with decreasing gestational age [2], [4], [5]. As expected, biological factors explained more of the variance in high compared with low or moderate cognitive workload tasks and in children born before 34 weeks of GA. Our results thus add further evidence that there is a curvilinear relationship between gestation and cognitive development with accelerating impact the lower the gestation [3], [27]. It is likely that inconsistent findings regarding the impact of moderate prematurity on cognitive abilities may be, at least partly, explained by use of tasks with different cognitive workload demands in different studies. Furthermore, although disproportionally higher deficits in performance were found for very and moderately preterm children our data additionally showed gradual cognitive deficits of late preterm compared with early term children (see Figure 1; 95% confidence intervals of mean value group comparisons: −.39 to −.07 for low cognitive workload, −.29 to −.04 for moderate cognitive workload, and −.36 to −.01 for high cognitive workload tasks, respectively). There were however no differences between early term and full term children's cognitive performance, irrespective of the workload of tasks.

Social influences, in addition to biological factors, are also important for general cognitive performance and frontal cortex development whether in healthy full term [43], [44] or preterm children [20], [28], [45]. There are different hypotheses of how social factors and parenting impact on cognitive performance. They may be equally important independent of the degree of biological risk or cognitive workload (additive model), i.e. have similar effects on cognitive performance in preterm and full term children [20], [43]. Alternatively, social factors and parenting may be particularly important when biological risk and workload are high (transactional model) [46], [47]. The aim of the present study was to operationalize and test a heuristic model that may explain the relationship between gestational age and cognitive performance – subsequently this cognitive workload model may be further scrutinized to understand the contributions of neuromotor abilities and the social environment to shape preterm children's cognitive abilities.

Our findings have important implications for neuroimaging research, routine follow-up, and intervention: Firstly, the validity of our cognitive workload model requires evaluation using not only functional cognitive performance but also neuroimaging data of the preterm brain. Particularly, Panigrahy and colleagues [48] have set the stage for developing a preterm connectome as a framework for future research. Accordingly, in order to scrutinize how the brain distributes its resources, activation and collaboration of cortical networks as a function of cognitive workload need to be investigated. For example, fMRI data would allow for testing of the hypothesis that functional interactions among intrinsic brain networks which are involved in goal-directed behaviour (i.e. the default mode and lateralized central executive networks [49]) are enlarged by increasing workload [50] and that this happens earlier or more often in preterm compared with healthy full term individuals. While this would indicate neuro-plasticity of brain organisation it comes at a cost for functional performance as shown here.

Secondly, routine cognitive follow-up of preterm children could benefit by organising assessments according to cognitive workload demands and thus provide a more detailed picture of the strengths and weaknesses of individual children and for planning their support. Thirdly, tentative evidence has emerged that adaptive computerized training can improve the working memory capacity of both full term [51], [52] and preterm children [53]. Accordingly, training-induced changes in both structural brain connectivity [54], [55] and brain activity in prefrontal and parietal networks [56], [57] have been found, which are attributed to increased working memory capacity [58]. In addition, it has been suggested that educational interventions could be developed in which information is not presented simultaneously to preterm children but more slowly and sequentially to promote academic attainment [59]. Our results support the merits of such an approach that would consider the cognitive workload requirements of educational tasks to plan lessons in school. For example, low cognitive workload tasks would provide reinforcement and successes for most learners whereas moderate and high cognitive workload tasks should be individually tailored for very and moderately preterm children who require a more adaptive and possibly slower transition. Research is needed to determine to which extend such interventions can influence children's developmental trajectories [60], [61].

### Strengths and limitations

The data were collected as part of a prospective geographically defined whole-population study of neonatal at-risk children and analyses were performed on a large population-representative sample. Detailed information on children's neonatal complications was available. At 8;5 years, cognitive assessments were carried out by trained assistant psychologists that were blind to children's background characteristics. The data set is based upon a cohort recruited in 1985/86. Medical and neonatal care has changed since then (i.e. introduction of corticosteroid and surfactant therapies), and has resulted above all in increased survival of ever lower gestation infants. However, rates of cognitive problems have remained at similar levels [62]. Thus more children survive while rates of cognitive problems have remained the same, thus overall more survivors will be in the community without but also with cognitive problems. Nevertheless, replications of our findings in more contemporary cohorts are needed.

Hypotheses were formulated according to a heuristic cognitive workload model. However, our findings are derived from a selective set of cognitive tasks. In future studies, the external validity of these findings for children's developmental and educational trajectories needs to be tested. Our model has been specifically designed to explain preterm children's cognitive performance; additional research may examine its validity for other at-risk populations (e.g. children with learning disabilities).

### Conclusion

The results support that cognitive performance of preterm children decreases as the cognitive workload of tasks increases. This relationship between gestation and task workload is curvilinear with disproportionally higher deficits in performance for very and moderately preterm children the higher the cognitive workload of the tasks. The cognitive workload model may help understand the association between task complexity and incremental performance deficits of preterm children and provides a heuristic framework for further research on neuro-plasticity. The findings have implications for organising cognitive follow-up of preterm children by providing more detailed feedback according to cognitive workload and for structuring both working memory and educational interventions.

## Supporting Information

Table S1 Sample characteristics according to gestational age groups (1) of the BLS Phase I total population sample (survivors up to 4;8 years of age, cases with severe neurological impairment excluded), (2) of the Phase II participants, and (3) of the Phase II participants weighted for neonatal biological risk.
(DOC)
