# Supplementary material for: Effects of Gestational Age at Birth on Cognitive Performance: A Function of Cognitive Workload Demands
Source: PLoS One. 2013 May 24;8(5):e65219. doi: 10.1371/journal.pone.0065219 (PMC3663809; doi:10.1371/journal.pone.0065219)
Supplement: Table S1 — Sample characteristics according to gestational age groups (1) of the BLS Phase I total population sample (survivors up to 4;8 years of age, cases with severe neurological impairment excluded), (2) of the Phase II participants, and (3) of the Phase II participants weighted for neonatal biological risk. (DOC) [file pone.0065219.s001.doc]

Table S1: Sample characteristics according to gestational age groups (1) of the BLS Phase I total population sample (survivors up to 4;8 years of age, cases with severe neurological impairment excluded), (2) of the Phase II participants, and (3) of the Phase II participants weighted for neonatal biological risk

|  | **< 32 w GA** | | **32-33 w GA** | **34-36 w GA** | **37-38 w GA** | **39-41 w GA** |
| --- | --- | --- | --- | --- | --- | --- |
| 1. **The geographically defined total population BLS Phase I sample** | | | | | | |
|  | ***n* = 372** | | ***n* = 427** | ***n* = 1737** | ***n* = 1545** | ***n* = 3694** |
| GA | 29.61 (1.56) | | 32.60 (0.49) | 35.17 (0.78) | 37.56 (0.50) | 39.89 (0.67) |
| Birth weight | 1313 (338) | | 1850 (348) | 2318 (420) | 2776 (547) | 3351 (545) |
| OPTI score | 9.74 (2.57) | | 7.18 (2.82) | 5.06 (2.53) | 3.77 (2.38) | 2.79 (2.23) |
| Ventilation (duration/days) | 18 (22) | | 3 (8) | 1 (3) | 0 (2) | 0 (2) |
| Clinic days | 82 (39) | | 44 (22) | 27 (20) | 17 (16) | 13 (16) |
| Neonatal risk score | | | | | | |
| none | 0% | | 0% | 3% | 11% | 18% |
| low | 0% | | 5% | 16% | 27% | 34% |
| moderate | 0% | | 25% | 39% | 38% | 32% |
| high | 0% | | 70% | 43% | 24% | 16% |
| very high | 100% | | 0% | 0% | 0% | 0% |
| 1. **The BLS Phase II sample** | | | | | | |
|  | ***n* = 255** | | ***n* = 90** | ***n* = 209** | ***n* = 186** | ***n* = 586** |
| GA | 29.55 (1.59) | | 32.52 (0.50) | 35.10 (0.76) | 37.54 (0.50) | 39.94 (0.68) |
| Birth weight | 1294 (348) | | 1656 (379) | 2207 (558) | 2828 (530) | 3393 (495) |
| OPTI score | 9.55 (2.66) | | 7.92 (2.60) | 5.48 (2.83) | 3.19 (2.66) | 1.95 (2.15) |
| Ventilation (duration/days) | 19 (23) | | 5 (9) | 1 (4) | 0 (2) | 0 (1) |
| Duration Clinic | 83 (41) | | 53 (22) | 29 (20) | 15 (16) | 11 (15) |
| Neonatal risk score | | | | | | |
| none | 0% | | 0% | 5% | 27% | 42% |
| low | 0% | | 2% | 17% | 27% | 25% |
| moderate | 0% | | 23% | 31% | 25% | 20% |
| high | 0% | | 74% | 46% | 21% | 13% |
| very high | 100% | | 0% | 0% | 0% | 0% |
| 1. **The weighted BLS Phase II sample** | | | | | | |
|  | ***n* = 255** | | ***n* = 90** | ***n* = 209** | ***n* = 186** | ***n* = 586** |
| GA | 29.55 (1.59) | | 32.52 (0.50) | 35.10 (0.76) | 37.52 (0.50) | 39.92 (0.67) |
| Birth weight | 1294 (348) | | 1656 (379) | 2207 (558) | 2809 (562) | 3364 (524) |
| OPTI score | 9.55 (2.66) | | 7.92 (2.60) | 5.48 (2.83) | 3.61 (2.53) | 2.63 (2.14) |
| Ventilation (duration/days) | 19 (23) | | 5 (9) | 1 (4) | 0 (2) | 0 (1) |
| Duration Clinic | 83 (41) | | 53 (22) | 29 (20) | 16 (16) | 12 (19) |
| Neonatal risk score | |  | | | | |
| none | 0% | | 0% | 5% | 14% | 17% |
| low | 0% | | 2% | 17% | 27% | 35% |
| moderate | 0% | | 23% | 31% | 38% | 34% |
| high | 0% | | 74% | 46% | 21% | 14% |
| very high | 100% | | 0% | 0% | 0% | 0% |
